# Satisfactory safety and immunogenicity of MSP3 malaria vaccine candidate in Tanzanian children aged 12–24 months

**DOI:** 10.1186/1475-2875-8-163

**Published:** 2009-07-17

**Authors:** John PA Lusingu, Samwel Gesase, Salum Msham, Filbert Francis, Martha Lemnge, Misago Seth, Samwel Sembuche, Acleus Rutta, Daniel Minja, Method D Segeja, Samuel Bosomprah, Simon Cousens, Ramadhani Noor, Roma Chilengi, Pierre Druilhe

**Affiliations:** 1 National Institute for Medical Research, Tanga Centre, Tanzania; 2 London School of Hygiene and Tropical Medicine, London, UK; 3 African Malaria Network Trust, Dar es Salaam, Tanzania; 4 Institut Pasteur Paris, Paris, France

## Abstract

**Background:**

Development and deployment of an effective malaria vaccine would complement existing malaria control measures. A blood stage malaria vaccine candidate, Merozoite Surface Protein-3 (MSP3), produced as a long synthetic peptide, has been shown to be safe in non-immune and semi-immune adults. A phase Ib dose-escalating study was conducted to assess the vaccine's safety and immunogenicity in children aged 12 to 24 months in Korogwe, Tanzania (ClinicalTrials.gov number: NCT00469651).

**Methods:**

This was a double-blind, randomized, controlled, dose escalation phase Ib trial, in which children were given one of two different doses of the MSP3 antigen (15 μg or 30 μg) or a control vaccine (Engerix B). Children were randomly allocated either to the MSP3 candidate malaria vaccine or the control vaccine administered at a schedule of 0, 1, and 2 months. Immunization with lower and higher doses was staggered for safety reasons starting with the lower dose. The primary endpoint was safety and reactogenicity within 28 days post-vaccination. Blood samples were obtained at different time points to measure immunological responses. Results are presented up to 84 days post-vaccination.

**Results:**

A total of 45 children were enrolled, 15 in each of the two MSP3 dose groups and 15 in the Engerix B group. There were no important differences in reactogenicity between the two MSP3 groups and Engerix B. Grade 3 adverse events were infrequent; only five were detected throughout the study, all of which were transient and resolved without sequelae. No serious adverse event reported was considered to be related to MSP3 vaccine. Both MSP3 dose regimens elicited strong cytophilic IgG responses (subclasses IgG1 and IgG3), the isotypes involved in the monocyte-dependant mechanism of *Plasmodium falciparum *parasite-killing. The titers reached are similar to those from African adults having reached a state of premunition. Furthermore, vaccination induced seroconversion in all vaccinees.

**Conclusion:**

The MSP3 malaria vaccine candidate was safe, well tolerated and immunogenic in children aged 12–24 months living in a malaria endemic community. Given the vaccine's safety and its induction of cytophilic IgG responses, its efficacy against *P. falciparum *infection and disease needs to be evaluated in Phase 2 studies.

## Background

Falciparum malaria remains a global health problem, accounting for 300–500 million clinical malaria episodes and estimated 1–3 million deaths annually [1]. About 90% of the burden occurs in sub-Saharan Africa, especially in children below five years of age [2]. Current tools to control malaria include use of insecticide-treated nets, intermittent preventive treatment in pregnancy and infants, and treatment on demand using effective anti-malarial drugs. An effective malaria vaccine would be an important complementary tool, whose development is considered to be a high priority [3].

There are three main categories of malaria vaccine candidates under research and development which target different phases of the malaria parasite's life cycle, namely pre-erythrocytic, blood stage and transmission blocking candidates[4]. It is well-established that malaria symptoms are associated with the erythrocytic stage of the life cycle and, therefore, the latter has attracted efforts to develop vaccines either to prevent invasion or to ensure parasite killing through antibody-triggered monocyte-mediators [5].

Whereas most vaccine candidates have been identified by experiments performed in experimental malaria models, Merozoite Surface Protein 3 (MSP3) is a candidate identified by clinical studies in humans. The passive transfer of protection by IgG from African adults into infected Thai children identified the co-operation of IgG with blood monocytes as the main defence mechanism in human beings, in an antibody-dependent, cellular inhibitory fashion (ADCI) [6]. Thereafter, the ADCI mechanism was used to screen a genome-wide expression library and identified MSP3 as the main target of antibodies mediating the monocyte-dependent *Plasmodium falciparum *killing effect [7]. The monocyte-dependent mechanism implies that only the cytophilic classes of IgG, namely IgG1 and IgG3, can act in the ADCI mechanism and epidemiological studies have confirmed that protection is associated with such cytophilic responses against MSP3 [8-11].

In the process of research and development, MSP3 as a long synthetic peptide, first underwent a Phase I trial in a malaria-naïve population, which demonstrated that the vaccine is safe and immunogenic, especially with aluminium hydroxide adjuvant compared to montanide adjuvant [12]. Moreover, antibodies elicited in volunteers mediated a very strong monocyte-dependent parasite killing effect [13]. Therefore, MSP3, adjuvanted with aluminium hydroxide, was further assessed in adults in a malaria endemic community in Burkina Faso, where it was found to be safe and able to elicit very significant immune responses even in individuals with pre-existing immunity [14].

This paper presents the results of a Phase Ib dose escalation trial among Tanzanian children aged 12–24 months that aim to assess the safety and immunogenicity of MSP3 adjuvanted by aluminium hydroxide when given at 0, one and two months schedule.

## Methods

### Study design

The safety and immunogenicity of either a 15 μg or a 30 μg dose of MSP3 in aluminium hydroxide adjuvant versus hepatitis B vaccine was assessed in a prospective double blind, randomized, dose escalation trial in 12 to 24 months old children in Korogwe, Tanzania (ClinicalTrials.gov identifier NCT00469651). The trial was conducted from November 2007 to November 2008. Ethical approval was granted by the Tanzania Medical Research Coordinating Committee (MRCC) and the ethical committee of the London School of Hygiene and Tropical Medicine (LSHTM), and regulatory approval was granted by the Tanzania Food and Drug Authority (TFDA). The design, conduct and results of the trial were overseen by a formally constituted data and safety monitoring board (DSMB) operating under a charter, an independent local safety monitor, and a clinical trial monitor. The study was conducted in compliance with Helsinki Declaration (1996 revision) and good clinical practice guidelines.

### Study site

The study was conducted in a lowland village at Korogwe District, north-eastern Tanzania. Korogwe District is about 100 kilometers inland from Tanga. Briefly, it is a tropical area with two rainy seasons: April to June and October to December. While January and February are normally dry, recently there have been climatic changes such that there is no clear distinction of these seasons. The estimated average entomological inoculation rate (EIR) ranged in the past from 30–100 infective bites per person per year [15,16], however this has decreased markedly in recent years. A demographic surveillance system funded by African Malaria Network Trust (AMANET) was established in the vaccination village in 2005. The study village has no primary health facility and Korogwe District Hospital, which is located 25 km away, serves the community. In lowland villages of Korogwe, malaria is perennial with peak seasons just after rain [15,17]. The lowland villages were characterized by high transmission, but the recent decline led to moderate transmission conditions (Lusingu *et al*, manuscript in preparation). In Korogwe District Hospital, malaria is the leading cause of admission and deaths among under five children [18].

### Participants

Healthy children by history and physical examination, whose parents or guardians gave voluntary, signed informed consent and were expected to be available for the whole study period of 12 months, were eligible for recruitment and enrolment if aged 12–24 months. The exclusion criteria included symptoms and physical signs of disease that could interfere with the interpretation of the trial results, or compromize the health of the subjects, immunosuppressive therapy (steroids, immune modulators or immune suppressors) within three months prior to recruitment (for corticosteroids, this meant prednisone, or equivalent, ≥ 0.5 mg/kg/day) and inability to be followed for any psychosocial or geographical reasons. Other criteria included laboratory abnormalities on screened blood samples, use of any investigational drug or vaccine other than the study vaccine within 30 days preceding the first dose of study vaccine, or planned use up to 30 days after the third dose, suspected or known hypersensitivity to any of the vaccine components or to previous vaccine, planned administration of a vaccine not foreseen by the study protocol within 30 days before the first dose of vaccine, an exception being the receipt of an EPI or licensed vaccine (measles, oral polio, Hepatitis B, meningococcal and combined diphtheria/pertussis/tetanus vaccines), which may be given 14 days or more before or after vaccination. Other exclusion criteria were evidence of chronic or active hepatitis B infection, presence of chronic illness that, in the judgement of the investigator, would interfere with the study outcomes or pose a threat to the participant's health, administration of immunoglobulin and/or any blood products within the three months preceding the first dose of study vaccine or planned administration during the study period, history of surgical splenectomy, and moderate or severe malnutrition at screening defined as weight for age Z-score less than -2.

### Study vaccines

The investigational vaccine was MSP3 and the control vaccine was hepatitis B (Engerix B). The anti-malaria vaccine MSP3 is a 96 amino-acids long synthetic peptide containing the amino-acid sequence 154–249 (3D7 clone). It is lyophilized with a white amorphous powder appearance. The vaccine is presented in multi-dose vials with the following formulation: active ingredient is MSP3 120 μg in sodium chloride 9 mg/mL, trisodium-citrate (10 mM) 2.94 mg/mL and disodium-phosphate buffer (10 mM) 1.42 mg/mL as manufactured by Synprosis Ltd, France. Before injection the vaccine is reconstituted in 0.9% saline solution and then reconstituted vaccine was then mixed with 1 mL of aluminium hydroxide, following the procedure recommended by the manufacturer, Synprosis Ltd, France. The minimum adsorption time of 60 minutes before injection was respected. Following reconstitution the vaccine was kept in a refrigerator between +2°C and +8°C temperature and used within six hours.

The adsorbed vaccine was aliquoted into single-use syringes, containing 15 μg or 30 μg of peptide. From one multi-dose vial, individual vaccine doses of 0.5 mL were prepared following a standard operating procedure (SOP) provided by the manufacturer. The vaccine was administered under sterile conditions, using a single-use syringe for each subject and following a strict procedure of disinfection of the injection site. The vaccine was administered subcutaneously.

Engerix B [Hepatitis B Vaccine (Recombinant)] is a non-infectious recombinant hepatitis B vaccine developed and manufactured by GlaxoSmithKline Biologicals. It contains purified surface antigen of the virus obtained by culturing genetically engineered *Saccharomyces cerevisiae *cells, which carry the surface antigen gene of the hepatitis B virus. The surface antigen expressed in *S. cerevisiae *cells is purified by several physicochemical steps and formulated as a suspension of the antigen adsorbed on aluminium hydroxide. The procedures used to manufacture Engerix B result in a product that contains no more than 5% yeast protein. No substances of human origin are used in its manufacture. Engerix B is supplied as a sterile suspension for intramuscular administration. The vaccine is ready for use without reconstitution and must only be shaken before administration.

### Study procedures

#### Site selection and sensitization

The study village was Kwashemshi village in Korogwe District, Tanga Region, Tanzania. The choice was based on data from the demographic surveillance system (DSS) according to which there were about 103 children between 12–24 months in Kwashemshi village.

Sensitization meetings were held with village leaders, elders and all villagers before beginning the study.

#### Screening and informed consent process

The flow of study participants during screening was based on a standard operating procedure. Briefly, there were seven stations that included registration, information giving, consent signing, clinical assessment, photo taking, blood draw and conclusion stations. Written informed consent in Kiswahili was obtained from parents or guardians during the screening visit. For those not able to write, consent was documented by thumbprint, with counter signature by a literate witness independent of the research team.

#### Administration of vaccines and evaluation of clinical parameters

Eligible children were randomly assigned to receive three doses of either MSP3 or Engerix B at intervals of 28 days. MSP3 vaccination was staggered such that following administration to the cohort receiving the 15 μg dose, a six-day follow-up was performed, after which a report was prepared and sent to Data Safety Monitoring Board (DSMB). Vaccination of children with 30 μg of MSP3-LSP only began once the DSMB had given written permission to proceed. The study was double blind in that neither the parents/guardians nor research team members involved with vaccination or clinical observation were aware of the vaccine allocation. Only staff responsible for preparing the vaccines were aware of the vaccine allocations, but these staff were not allowed any other role in the study.

Enrolled children were vaccinated by subcutaneous injection on days 0, 28 and 56. On each vaccination visit, children were assessed clinically and 3–5 mls of venous blood was collected for safety and immunogenicity parameters, before administration of the study vaccines. Appropriate medical treatment kit in case of an immediate anaphylactic reaction following the administration of the vaccine was always available during vaccine administration.

Immediate reactogenicity was observed for one hour following each vaccine dose. At all vaccination sessions, a local safety monitor was available at the vaccination centre. Following each vaccination, all children were visited at home by a field worker for six days to evaluate solicited local symptoms (pain, swelling, induration, erythema and pruritus at injection site); and solicited systemic reactions, including fever (defined as axillary temperature ≥ 37.5°C), drowsiness, loss of appetite and irritability/fussiness (Table 1). Unsolicited adverse events occurring within 28 days following each vaccination and serious adverse events (SAE) occuring at any time during the trial period (from first visit to last visit) were also documented. Parents and guardians were encouraged to contact field workers if their child was ill.

**Table 1:** Severity grading of solicited adverse events

**Adverse Event**	**Intensity grade**	**Parameter**
Pain at injection site	0	Absent
	1	Minor reaction to touch
	2	Cries/protests on touch
	3	Cries when limb is moved/spontaneously painful
Swelling at injection site		Recorded greatest surface diameter in mm
	0	None
	1	< 5 mm
	2	5 to 20 mm
	3	> 20 mm
Induration at injection site		Recorded greatest surface diameter in mm and graded as swelling above
Erythema at injection site		Recorded greatest surface diameter in mm and graded as swelling above
Pruritus at injection site	0	Absent
	1	Easily tolerated by the subject, causing minimal discomfort and not interfering with everyday activities.
	2	Sufficiently discomforting to interfere with normal everyday activities.
	3	Prevents normal, everyday activities.
Fever		Recorded axillary temperature in °C
	0	< 37.5°C
	1	37.5 °C – 38.0°C
	2	> 38°C – 39.0°C
	3	> 39.0°C
Irritability/Fussiness	0	Behavior as usual
	1	Crying more than usual/no effect on normal activity
	2	Crying more than usual/interferes with normal activity
	3	Crying that cannot be comforted/prevents normal activity
Drowsiness	0	Behavior as usual
	1	Drowsiness easily tolerated
	2	Drowsiness that interferes with normal activity
	3	Drowsiness that prevents normal activity
Loss of appetite	0	Appetite as usual
	1	Eating less than usual/no effect on normal activity
	2	Eating less than usual/interferes with normal activity
	3	Not eating at all

### Measurement of biological parameters

Biological variables measured included haematological [Hb (g/dl)], white blood cells [WBC (10,000/μl)], platelets (1,000/μl), and biochemical [alanine aminotransferase ALT (IU/l), creatinine (μmol/l) and bilirubin (mol/l)] parameters. Biochemical tests were run with serum or plasma using a dry enzymatic detection system with a Vitros DT60 II analyser (Orthoclinical Diagnostics, Johnson and Johnson, Rochester, NY, USA). This system is based on independent slides for each biochemical parameter with all the necessary reagents for enzymatic reactions incorporated. Three modules were used, chosen depending on the parameter to be analysed: DT60II Chemistry System, DTE II Module and DTSC Module.

Haematological tests were conducted using a Sysmex KX-21N cell counter (Kobe, Japan), which determines blood parameters using total blood in EDTA. The KX-21N has three detector blocks: the white blood cell (WBC) detector block, the red blood cell (RBC) detector block and the haemoglobin (Hb) detector block. The WBC detector block counts leukocytes using the direct current resistance (DCR) method (determining the changes of electric impulses between two electrodes where the diluted sample is circulating). Erythrocyte and platelet counts are performed in the RBC detector block, also by the DCR method. The third block (HGB detector block) measures the haemoglobin concentration using the no-cyanide Hb method. All laboratory procedures adhered to good laboratory practices following established standard operational procedures (SOPs).

### Measurement of antibody responses

Anti-MSP3 specific antibodies (total IgG, IgM and of each of the four IgG subclasses) were measured using indirect enzyme-linked immunosorbent assay (ELISA) [19]. All antigens tested were optimized and shown to be stable for at least three weeks, when antigen-coated plates and serum/plasma dilutions are refrigerated. The subclass specific reagents used were selected on the basis of low cross reactivities among themselves, and ability to faithfully react with African heavy chains dominant allotypes [19]. To control for inter-assay and day-to-day variations in the standardized ELISA procedure, three-fold serial dilutions of reference standard reagents (IgG, IgM and IgG1 to IgG4) were directly coated onto each ELISA plate (Maxisorp Nunc, Denmark) at a start concentration of 1,000 ng/ml (100 μl/well).

Optical density values for the test samples were converted into antibody units with the standard reference curves generated for each ELISA plate using a four parameter curve-fit Microsoft Excel-based application. Samples were re-tested if the coefficient of variation between duplicate absorbance values were higher than 15% and plates were also re-tested if the R-squared value of the standard curve was less than 97%. The reference standards, PBS buffer blank, positive and negative control plasma pools that were included in each ELISA test plate allow for the determination of failed assay runs. The Afro-Immuno-Assay network ELISA procedure used in this study is described in detail at the AMANET website . Although all children received all three vaccine doses as per protocol, the number of children whose blood samples were available in sufficient amount to evaluate anti-MSP3 antibodies at day 84 were 15/15 in the 15 μg dose of MSP3, 12/15 in the 30 μg dose of MSP3 and 13/15 in Engerix B group. Results are shown below for those individuals whose sera were available at day 84.

In order to compare immunogenicity data obtained in African infants with previous immunizations performed in European volunteers and immune responses following exposure to high level of transmission, the sera of the 6 European volunteers immunized previously in Phase 1a in Lausanne with 30 micrograms of MSP3 adjuvanted by alum were tested in parallel with six randomly chosen sera from the cohort followed in Korogwe (irrespective of the dose of MSP3 received). The pool of 200 African adults from a rural area of Ivory Coast that was employed in passive transfer in Thai subjects and 4 adults from the village of Dielmo where malaria transmission is one of the highest in the world, with an average 250 infective bites per person per year were used as positive controls[10]. Results were expressed in AU as described in [19].

### Data management

Participant information was captured on paper case report forms (CRFs), which were double-entered into a Microsoft Access data base. The cleaned and validated database was then analysed using STATA version10 , based on a pre-defined analysis plan. Safety and reactogenicity analyses included all randomized children for whom any safety data were available, and comprised descriptive statistics. The primary measures of safety and reactogenicity were the frequency of local and systemic solicited adverse reactions for six days after each dose and the frequency of unsolicited adverse events, up to 28 days after each dose. The final descriptive analysis was planned one month after dose 3 to evaluate the safety and immunogenicity. The trial sample size was determined on the primary evaluation criteria, concerning the occurrence of systemic adverse events. The total sample size was calculated at 45 children to be distributed as: 15 to receive 15 μg MSP3 vaccine, 15 to receive 30 μg MSP3 vaccine and 15 to receive Engerix B vaccine, allowing for losses to follow-up or protocol deviations of not more than 10%. With 14 children completing follow-up in each MSP3 arm, the study would have 90% power to detect at least one MSP3 vaccinated individual with a systemic reaction (or a serious adverse event) if the underlying risk of such an event was 15% or more. The trial would have 80% power to detect at least one individual if the underlying risk was 11% or more and 95% power to detect at least one individual if the underlying risk was 19% or more. Immunogenicity focused primarily on antibody responses. Unblinding was done at Tanga Centre, attended by the sponsor, inventor, LSM and the investigating team.

## Results

### Participant distribution and demographic data

**Figure 1:**
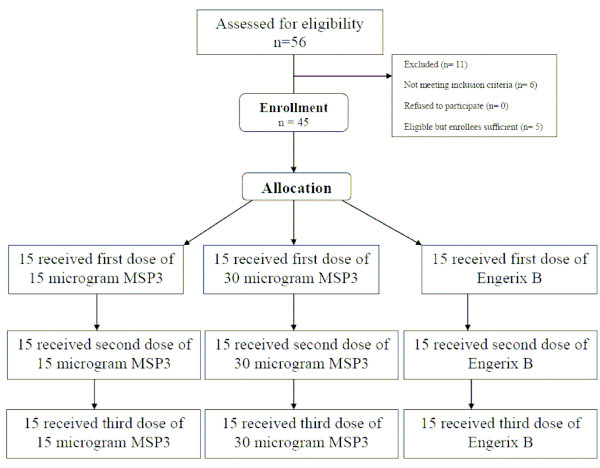
MSP3 Phase 1 trial flow diagram.

A total of 45 children 12–24 months old were enrolled and randomized, 15 to each treatment group (Figure 1). All enrolled children were included in the safety population since 100% (45/45) of children received all three vaccinations and all children completed the day 84 follow-up visit. The median age of children was 1.4 years (minimum 1.0 year and maximum 2.0 years) with approximately equal numbers of males and females.

### Safety and reactogenicity

Both MSP3 vaccine doses and Engerix B vaccine formulations were well tolerated. The incidence of local and systemic solicited reactions is shown in Table 2 and Table 3, respectively. The only major difference observed was for induration among MSP3 vaccinated children compared with Engerix B vaccinated children (Fisher's exact p-value = 0.07). None of the grade three solicited symptoms were classified as likely to be related to the vaccines.

**Table 2:** Incidence of solicited local adverse events per vaccine type per dose

		**First dose**						**Second dose**						**Third dose**					
		**15 μg MSP3**		**30 μg MSP3**		**Engerix B**		**15 μg MSP3**		**30 μg MSP3**		**Engerix B**		**15 μg MSP3**		**30 μg MSP3**		**Engerix B**	
		**N = 15**		**N = 15**		**N = 15**		**N = 15**		**N = 15**		**N = 15**		**N = 15**		**N = 15**		**N = 15**	
**Symptom**	**Intensity**	**n**	**%**	**n**	**%**	**n**	**%**	**n**	**%**	**n**	**%**	**n**	**%**	**n**	**%**	**n**	**%**	**n**	**%**
**Any**	Overall	8	53	10	67	7	47	12	80	6	40	6	40	9	60	12	80	9	60
	Grade 3	0	0	0	0	0	0	0	0	0	0	0	0	0	0	0	0	0	0
																			
**Pain**	Overall	3	20	2	13	1	6.7	1	6.7	1	6.7	1	6.7	0	0	1	6.7	2	13
	Grade 3	0	0	0	0	0	0	0	0	0	0	0	0	0	0	0	0	0	0
																			
**Swelling**	Overall	3	20	7	47	5	33	4	27	1	6.7	1	6.7	3	20	2	13	3	20
	Grade 3	0	0	0	0	0	0	0	0	0	0	0	0	0	0	0	0	0	0
																			
**Erythema**	Overall	0	0	2	13	1	6.7	1	6.7	1	6.7	1	6.7	1	6.7	0	0	2	13
	Grade 3	0	0	0	0	0	0	0	0	0	0	0	0	0	0	0	0	0	0
																			
**Induration**	Overall	8	53	10	67	6	40	**12**	**80**	**7**	**47**	**6**	**40**	8	53	12	80	8	53
	Grade 3	0	0	0	0	0	0	0	0	0	0	0	0	0	0	0	0	0	0
																			
**Pruritus**	Overall	0	0	0	0	0	0	0	0	0	0	1	6.7	0	0	0	0	0	0
	Grade 3	0	0	0	0	0	0	0	0	0	0	0	0	0	0	0	0	0	0

**Table 3:** Incidence of solicited systemic adverse events per vaccine type per dose

		**First dose**						**Second dose**						**Third dose**					
		**15 μg MSP3**		**30 μg MSP3**		**Engerix B**		**15 μg MSP3**		**30 μg MSP3**		**Engerix B**		**15 μg MSP3**		**30 μg MSP3**		**Engerix B**	
		**N = 15**		**N = 15**		**N = 15**		**N = 15**		**N = 15**		**N = 15**		**N = 15**		**N = 15**		**N = 15**	
**Symptom**	**Intensity**	**n**	**%**	**n**	**%**	**n**	**%**	**n**	**%**	**n**	**%**	**n**	**%**	**n**	**%**	**n**	**%**	**n**	**%**
**Any**	Overall	6	40	6	40	5	53	1	6.7	0	0	2	13	0	0	1	6.7	0	0
	Grade 3	0	0	0	0	0	0	0	0	0	0	0	0	0	0	0	0	0	0
	Related	0	0	0	0	0	0	0	0	0	0	0	0	0	0	0	0	0	0
																			
**Fever**	Overall	2	13	2	13	2	13	1	6.7	0	0	2	13	0	0	1	6.7	0	0
	Grade 3	0	0	0	0	0	0	0	0	0	0	0	0	0	0	0	0	0	0
	Related	0	0	0	0	0	0	0	0	0	0	0	0	0	0	0	0	0	0
																			
**Irritability**	Overall	2	13	0	0	2	13	0	0	0	0	0	0	0	0	0	0	0	0
	Grade 3	0	0	0	0	0	0	0	0	0	0	0	0	0	0	0	0	0	0
	Related	0	0	0	0	0	0	0	0	0	0	0	0	0	0	0	0	0	0
																			
**Drowsiness**	Overall	1	6.7	1	6.7	1	6.7	0	0	0	0	0	0	0	0	0	0	0	0
	Grade 3	0	0	0	0	0	0	0	0	0	0	0	0	0	0	0	0	0	0
	Related	0	0	0	0	0	0	0	0	0	0	0	0	0	0	0	0	0	0
																			
**Loss of appetite**	Overall	5	33	5	33	0	0	0	0	0	0	0	0	0	0	0	0	0	0
	Grade 3	0	0	0	0	0	0	0	0	0	0	0	0	0	0	0	0	0	0
	Related	0	0	0	0	0	0	0	0	0	0	0	0	0	0	0	0	0	0

There were five children who had serious adverse events (SAEs) reported and one of these was classified as likely to be related the control Engerix B vaccine. This was a febrile episode that led to hospitalization a day after the first dose of Engerix B, and whose aetiology remains unclear. Urine analysis was normal. There were no signs of pneumonia and no focal point of the fever was established. Malaria parasites were not found in a blood smears. The child was treated with antibiotics and antipyretics. The febrile episode resolved and the child was discharged within five days, with no sequelae. The child received second and third vaccination doses without further adverse events detected. The other four children had SAEs that included pneumonia and acute gastroenteritis. One of these children was vaccinated with 30 μg MSP3, two of the children were vaccinated with 15 μg MSP3, and one was vaccinated with Engerix B. None of these events were deemed to be causally-related to investigational or control vaccines.

**Figure 2:**
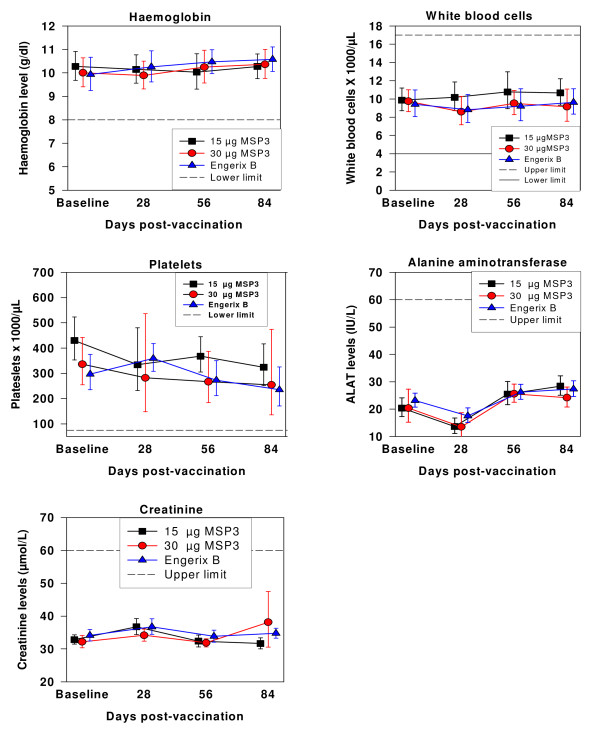
**Haematology and biochemical safety parameters**. Line with filled squares show children vaccinated with 15 μg MSP3, filled circles show children vaccinated with 30 μg MSP3 and filled triangles show children vaccinated with Engerix B. Error bars show 95% confidence interval.

At baseline, all children had haematological and biochemical parameters within the normal ranges (Figure 2), and this remained the case for all three vaccine groups throughout the study period.

### Immunogenicity

**Figure 3:**
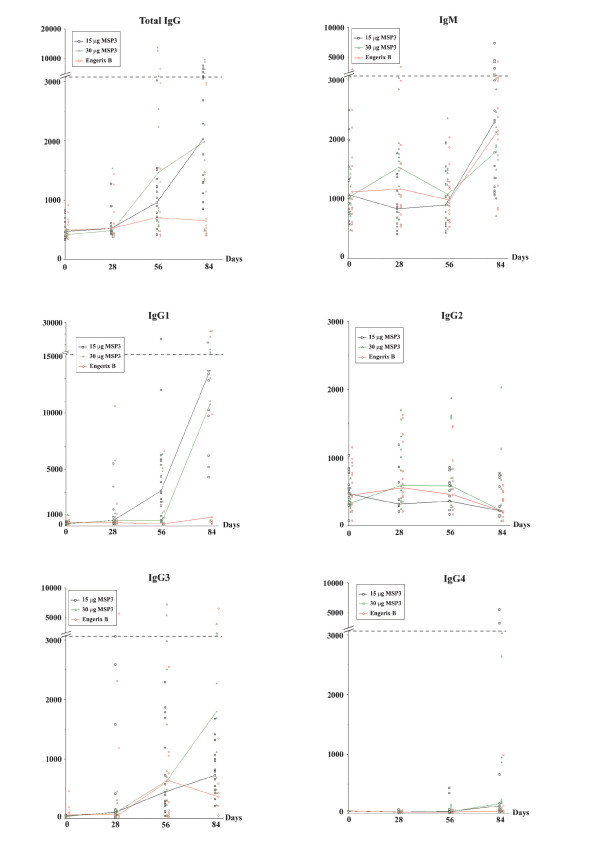
**Individual pattern of antibody responses to MSP3 by vaccination dose with respect to vaccine type**. Squares show children vaccinated with 15 μg MSP3, triangles show children vaccinated with 30 μg MSP3 and circles show children vaccinated with Engerix B. Lines connect median values. Abscissas: days post vaccination and ordinates: Elisa titers.

**Figure 4:**
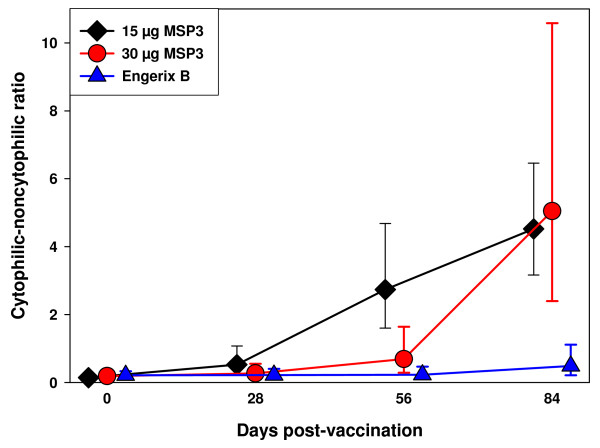
**The pattern of cytophilic to non-cytophilic ratio**. Line with filled squares show children vaccinated with 15 μg MSP3, filled circle show children vaccinated with 30 μg MSP3 and filled triangle show children vaccinated with Engerix B. Error bars show 95% confidence interval.

Anti-MSP3 antibodies were measured at baseline, 28 days, 56 days and 84 days. At baseline, before immunization all children had very low levels of anti-MSP3 antibodies in both the vaccine and control groups (Figure 3). Anti-MSP3 total IgG increased after the second and the third immunizations in both vaccinated groups, whereas no major change was seen in the Engerix B controls. Similar increases were observed in the 15 and the 30 μg dose groups of MSP3. Isotype studies of the distribution of anti-MSP3 cytophilic (IgG1+IgG3) versus noncytophilic [IgG2+IgG4+IgM)] classes provided very clear-cut results. Baseline values were similarly low among all children. IgG1 antibodies showed a large increase in concentrations, after the second and mostly after the third immunization, with no major difference between 15 or 30 μg of MSP3. No such increase was observed in the control group. The other cytophilic antibody class (IgG3) showed a marked increase after the second dose in both MSP3 dose groups, although there was some indication of an increase in the control group after the third dose. IgM concentrations increased moderately after the third immunization, by two-fold only, and as a similar increase was seen among individuals receiving the control vaccine EngerixB, the antigen-specificity of this result can be questioned (eg. this increase may be the result of a polyclonal activation due to the adjuvant). The proportion of study participants with an 8-fold increase in anti-MSP3 IgG subclasses at day 84 relative to their baseline status was the highest for IgG1 and IgG3 antibodies at both MSP3 vaccine doses (Table 4). This almost exclusive dominance of the cytophilic classes IgG1 and IgG3 is graphically expressed by the high cytophilicity ratio shown in Figure 4.

**Table 4:** Proportion of children with 8-fold increase of anti-MSP3 antibodies at day 84 by vaccine type

**Antibody**	**15 μg MSP3**			**30 μg MSP3**			**Engerix B**		
	
	**N**	**n**	**%**	**N**	**n**	**%**	**N**	**n**	**%**
**Total IgG**	15	4	26.7	12	4	33.3	13	1	7.7
**IgG1**	15	15	100	12	10	83.3	13	2	15.4
**IgG2**	15	0	0	12	0	0	13	0	0
**IgG3**	15	13	86.7	12	11	91.7	13	6	46.2
**IgG4**	15	3	20	12	4	33.3	13	1	7.7

N = total vaccinated, n = responders

Results from comparative immuno-assays with European Phase Ia volunteers sera, and adult sera from hyperendemic areas, show that only four of the six European volunteers sero-converted, two being negative following immunization and the remaining four having a mean titre ± SD of 6.12 ± 1.66, whereas all infants studied in Tanga seroconverted and among the six included in the comparative analysis, the mean titre + SD was of 10.68 ± 4.64 (Figure 3). This compares very well with the pool of Ivory Coast adults which ELISA titre was of 11.48 and the mean + SD of four adults from the high transmission area of Dielmo which titre was of 13.65 ± 3.63.

## Discussion

The results indicate that MSP3 adjuvanted with aluminium hydroxide is safe and has an acceptable reactogenicity profile when administered according to 0, 1, 2-month vaccination schedule to children aged 12–24 months in Tanzania. This is consistent with other studies that have also shown that MSP3 is safe in naïve and non-naïve adults [12,14]. The reactogenicity of both doses of MSP3 appeared to be similar to Engerix B. Adverse events including SAEs were infrequent and all resolved within few days without sequelae. One SAE, which was likely related to vaccination, occurred in the group that was vaccinated with Engerix B. There was no significant difference in haematology or biochemical parameters between vaccine groups.

The design of this study had some inherent weakness for evaluating reactogenicity. Thus the observed high incidence of induration may be related to the fact that MSP3 was administered subcutaneously (SC), while the control vaccine was given intramuscularly. The reason for choosing the SC route was that the safety and immunogenicity of the vaccine had been previously documented by this route under Phase Ia, and Ib. Considerations should be given to evaluating MSP3 via intramuscular route to be consistent with the route generally used for other vaccines in the expanded programme on immunization.

Some humoral immune responses to MSP3 were markedly higher in both MSP3 dose groups as compared to Engerix B vaccination. Most importantly all individuals receiving the vaccine developed specific immune responses and the antibodies produced were almost exclusively in the two classes IgG1 and IgG3 which can bind Fcγ Receptors and trigger the monocyte-dependant anti-parasite killing effect [5]. Transmission intensity being relatively low in this area of Tanzania, most children probably had little or no previous exposure to malaria. Hence these results show that a very simple formulation of MSP3 adjuvanted by alum, the adjuvant with the widest experience and the most affordable, can induce in young African children anti-MSP3 antibodies at high titers, predominantly if not solely made of the IgG subclasses found most effective in protecting against malaria [5,10,20]. Such an antibody profile is just what is required. Further studies are on-going to define the biological anti-parasite activity of those antibodies under *in-vitro *conditions, although it is clear that only Phase II can define their true protective effects.

Comparative immunological results obtained in Korogwe indicate very satisfactory immunogenicity with induction of MSP3 specific antibodies in all children receiving the vaccine. This is in contrast with results obtained previously at the 30 μg dose in European adult volunteers, not all of whom seroconverted [5,10,20]. In addition, the titres induced in young children were far higher than those elicited in European volunteers and essentially similar to those found in African adults exposed since birth to continuous sporozoite challenge and multiple blood stage infections. The children with limited prior exposure to natural infection had baseline values before immunization that were essentially similar to those of non-immune Europeans. In other words, the MSP3 vaccine induced an antibody response in 12 – 24 months old children similar to that seen in adults exposed for more than 20 years to the MSP3 protein presented by the parasite.

This was the first Phase 1 malaria vaccine trial to be performed in Tanzania and some lessons were learnt during the course of the trial. First, communities were well aware and eager to participate in the process of development and deployment of a malaria vaccine. This is to be expected given that malaria is a leading cause of morbidity and mortality in children, as well being responsible for economic underdevelopment [21]. Second, the concept of investigating the safety of a malaria vaccine is understood with difficulty by the community. Strenuous efforts were needed to explain the concept of safety. Third, the involvement of African based sponsors (AMANET) for malaria vaccines and the involvement of African researchers (NIMR) in leading these malaria vaccine trials will develop capacity of researchers from malaria endemic regions in not only conducting vaccine trials but also come up with strategies that will foster new ideas in conducting successful clinical trials.

In conclusion, the results have confirmed that MSP3 is safe and immunogenic in children and highlights the need to conduct Phase 2 trials to evaluate the efficacy of MSP3 in children living in malaria endemic communities. Since 15 μg and 30 μg of MSP3 vaccine doses were similarly well tolerated, it is reasonable to recommend that the higher dose be taken forward for evaluation of efficacy.

## Competing interests

The authors are not aware of any issue that would constitute a conflict of interest in the work done and results presented in this paper.

## Authors' contributions

The investigational team (JPAL, SG, SM, FF, MN, MS, SS, AR, DM, MML and MDS), were involved in study design, implementation of the trial, collection and analysis of data and manuscript writing. The study sponsor staff (RC, RN), were involved in the study design, monitoring of the study, and writing of the manuscript. The vaccine inventor (PD) was involved in trial design and writing of the manuscript. Collaborators from the London school (SB, SC) were involved in the study design, sample size calculations, analysis of results and manuscript writing. All authors had full access to data after database lock. The corresponding author (JL) drafted the manuscript and had final responsibility for the decision to submit this manuscript for publication. All authors read and approved the final manuscript.
